# The role of first-trimester HbA1c in the early detection of gestational diabetes

**DOI:** 10.1186/s12884-021-04330-2

**Published:** 2022-01-27

**Authors:** Mehrnaz Valadan, Zeinab Bahramnezhad, Fatemeh Golshahi, Elham Feizabad

**Affiliations:** 1 Department of Obstetrics and Gynecology, Yas Hospital, Tehran University of Medical Sciences, Tehran, Iran; 2 Maternal, Fetal and Neonatal Research Center, Tehran University of Medical Sciences, Yas Hospital, Tehran, Iran

**Keywords:** Gestational diabetes mellitus, OGTT, HbA1c, Screening

## Abstract

**Background:**

We aimed to assess the utility of HbA1c in the early detection of gestational diabetes (GDM) in the first trimester.

**Methods:**

This prospective study was performed on 700 pregnant women in the perinatology clinic at a tertiary university hospital from March 2018 to March 2020. For all pregnant women, HbA1c and fasting blood glucose (FBG) levels were examined during the first trimester. Then, a GDM screening test was done within 24–28 weeks of pregnancy using a 100 g oral glucose tolerance test (OGTT) as the gold standard test. The GDM diagnosis was made according to the American Diabetes Association (ADA) criteria. Sensitivity, specificity, positive (PPV), and negative predictive value (NPV) of HbA1c and FBG were calculated using the receiver operating characteristic (ROC) curve.

**Results:**

Of 700 participants, one hundred and fifteen (16.4%) women had GDM. The GDM patients were significantly older and had a higher pre-gestational body mass index and pregnancy weight gain compared to the non-GDM participants. The sensitivity and specificity for ruling out GDM at an HbA1c cut-off value of 4.85% was 92.2 and 32.8%, respectively, with a 95.5% NPV and a 21.2% PPV. Furthermore, sensitivity and specificity for diagnosing GDM at an HbA1c cut-off value of 5.45% was 54.8 and 96.8%, respectively, with a 91.5% NPV and a 76.8% PPV. Using HbA1c could decline OGTT in 40.4% of the pregnant women (28.7% with HbA1c < 4.85 and 11.7% with HbA1c ≥ 5.45%).

**Conclusion:**

It seems that the first-trimester HbA1c cannot replace OGTT for the diagnosis of GDM because of its insufficient sensitivity and specificity. However, women with higher first-trimester HbA1c had a high risk for GDM incidence.

## Introduction

Gestational diabetes mellitus (GDM) or diabetes mellitus in pregnancy is the most prevalent metabolic abnormality during pregnancy and is defined as diabetes first detected at any time during pregnancy [1, 2].

The prevalence of GDM is on the rise since the past decades, with an overall frequency of 17.8% (range 9.3–25.5%) [3]. The possible causes for this enhancement are the rise in maternal age and body mass index (BMI), access to the prenatal screening test, and changes in diabetes diagnostic thresholds [2].

GDM can cause severe obstetrics complications, which affect both mothers and their offspring. A higher risk of preeclampsia and cesarean section rate, fetal growth abnormalities, macrosomia prematurity, neonatal hypoglycemia, as well as maternal and neonatal trauma are some common examples of GDM [4, 5].

Former evidence indicated some important risk factors for GDM in pregnant women, including BMI > 38.6 kg/m^2^, fasting glucose > 4.5 mmol/L (about 81 mg/dL), abdominal circumference > 91.5 cm, and presence of polycystic ovary syndrome (PCOS). Moreover, there is a significant increase in the GDM incidence if some of these risk factors coexist simultaneously [6].

Among the abovementioned risk factors, fasting glucose (FG) of 5.6 to 7 mmol/L, at the first prenatal visit was strongly associated with some adverse pregnancy outcomes such as shoulder dystocia/birth injury (OR 24.5; 95% CI: 2.8–214.8), and preeclampsia (OR 2.7; 95% CI: 1.2–5.9) even after adjustment for maternal age and BMI [7].

The oral glucose tolerance test (OGTT) is a well-known screening test for the diagnosis of GDM in pregnant women. However, the deficiencies of this test include the need for eight-hour fasting, obtaining at least two blood samples, vomiting, and its high variability. Hence, about 10% of pregnant women fail to complete their OGTT process [8–11].

Glycosylated hemoglobin (HbA1c) is mainly used to detect and manage type 2 diabetes mellitus. Compared to FBS and OGTT, the HbA1c test shows the two to three previous months’ mean glucose concentration. Fasting is not required for this measurement and has a lower intra-individual variability, which causes the test to be more acceptable for the patients [12, 13].

Although it seems HbA1c is an easy, acceptable, and helpful lab test in GDM diagnosis, first-trimester HbA1c usefulness to detect women who will develop GDM has not been evaluated sufficiently, and it has not been mentioned yet in any of the current guidelines for GDM [5, 14, 15].

Therefore, we aimed to assess the diagnostic profile of the first-trimester HbA1c in the early detection of GDM by determining the cut-off levels for ruling out and diagnosing first-trimester GDM.

## Method

This prospective study was included 760 pregnant women who presented for their regular pregnancy care to the perinatology clinic at a tertiary university hospital, Yas hospital, from March 2018 to March 2020.

All singleton pregnant women older than 18 years, referred to our clinic within their first trimester of pregnancy (by the end of the 12th week of their gestational age), were included in this study. Pregnant women with type I or II diabetes mellitus, multiple pregnancies, spontaneous abortions, and elective termination of pregnancies were excluded. Furthermore, the patients who withdrew at any phase during the study were excluded. Forty women were excluded because of type II diabetes mellitus and 20 women withdrew to participate in the study.

A detailed history of all the patients was recorded at baseline. The first trimester HbA1c and fasting blood glucose (FBG) levels were requested for all participants. For measuring FBG, a venous blood sample was obtained from every participant in the morning and after 9 h of fasting. FBG was determined using commercially available laboratory kits via enzymatic methods and spectrophotometry techniques.

Between the 24th and 28th week of gestation, a Glucose Challenge Test (GCT) (blood glucose one hour post-50-g glucose without fasting) was requested for all participants. All tests were performed in the laboratory of Yas Hospital. In participants with abnormal blood glucose levels (GCT ≥140), OGTT with 100 g glucose after 8–10 h of overnight fasting was requested, and blood glucose levels were measured before and 1, 2, and 3 h after administration of 100 g of glucose.

The GDM diagnosis was made according to American Diabetes Association (ADA) criteria (having two or more plasma glucose levels higher than these cut-offs), FBS ≥ 95 mg/dl, BS one hour after 100 g glucose ≥180 mg/dl, BS two hours after 100 g glucose≥155 mg/dl, BS three hours after 100 g glucose ≥140 mg/dl [13].

Women diagnosed with GDM, overt diabetes (HbA1c ≥ 6.5% or/and FBS ≥126 mg /dl) at any time of our study were referred to an endocrinologist for immediate counseling and treatment. The women with an HbA1c < 6.5% received no extra treatment or additional testing. In addition, anemia was defined as a hemoglobin level less than 11 g/dl.

### Statistical analysis

The data were analyzed with the statistical software package IBM SPSS Statistic version 24.0. The quantitative variables were presented as mean ± standard deviation and the categorical variables as frequency (percentages). The quantitative variables with normal distribution were compared between the groups using an independent T-test, and the Chi-square test was used to compare the categorical variables. Receiver operating characteristic (ROC) curve was applied for sensitivity, specificity, positive (PPV), and negative predictive value (NPV) calculation of distinct first-trimester HbA1c cut-off.

## Results

**Fig. 1:**
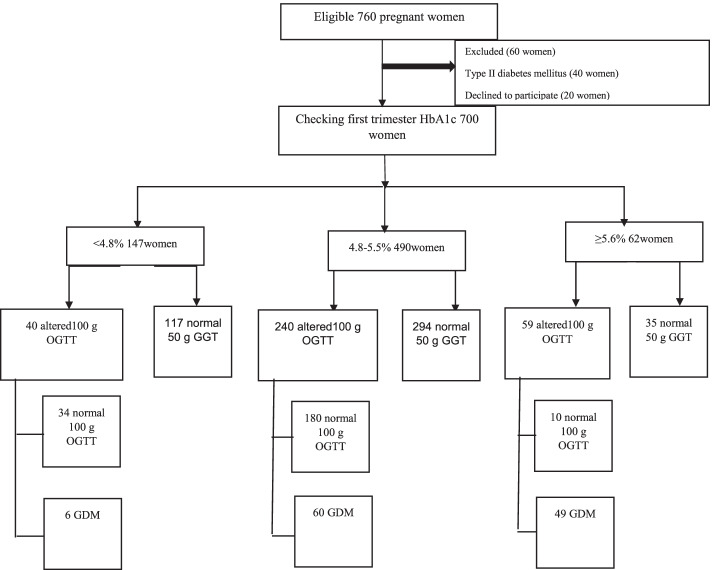
Flow chart of the study protocol. OGTT: Oral Glucose Tolerance Test. GDM: Gestational Diabetes Mellitus

Of 700 participants, 115 (16.4%) women were diagnosed with GDM during the study (Fig. 1). Women with GDM had significantly older age, higher pre-gestational body mass index (BMI), and pregnancy weight gain compared to the non-GDM pregnant women. All the baseline characteristics of the pregnant women are summarized in Table 1.

**Table 1:** The study population’s information

Variables	GDM	No GDM	***P*** value
	(***n*** = 115)	(***n*** = 585)	
**Age, years**	32.64 ± 5.49	30.64 ± 5.17	< 0.001
**Gestational age, weeks**	10.2 ± 2.05	10.64 ± 1.66	0.033
**Pre-pregnancy BMI, kg/m2**	27.23 ± 3.93	24.89 ± 2.93	< 0.001
**Pregnancy weight gain, kg**	10.28 ± 2.3	9.64 ± 1.53	0.005
**1st trimester FBG, mg/dl**	92.01 ± 7.79	82.61 ± 6.46	< 0.001
**1st trimester HbA1c, %**	5.45 ± 0.39	4.96 ± 0.30	< 0.001
**Hemoglobin, g/dl**	13.05 ± 0.85	13.06 ± 0.96	0.938
**MCV, fl**	84.11 ± 4.53	84.40 ± 3.41	0.516
**Previous GDM**	14 (12.1)	8 (1.3)	< 0.001
**Family history of DM**	53 (46.1)	122 (20.8)	< 0.001
**Previous macrosomia**	6 (5.2)	5 (0.8)	0.001
**Physical activity**			
Bed rest	0 (0)	4 (0.7)	0.003
Inter home activity	73 (63.5)	274 (46.8)	
Extra home activity	42 (36.5)	307 (52.5)	
**PCO/Metabolic syn.**	32 (27.8)	38 (6.4)	< 0.001
**Anemia**	12 (10.4)	59 (10.1)	0.910
**Birth weight, g**	3519.13 ± 301.91	3307.06 ± 192.46	< 0.001

*GDM* gestational diabetes mellitus, *BMI* body mass index, *FBG* fasting blood glucose, *HbA1c* glycosylated hemoglobin, *MCV* mean corpuscular volume, *PCO* Polycystic ovary syndrome

**Fig. 2:**
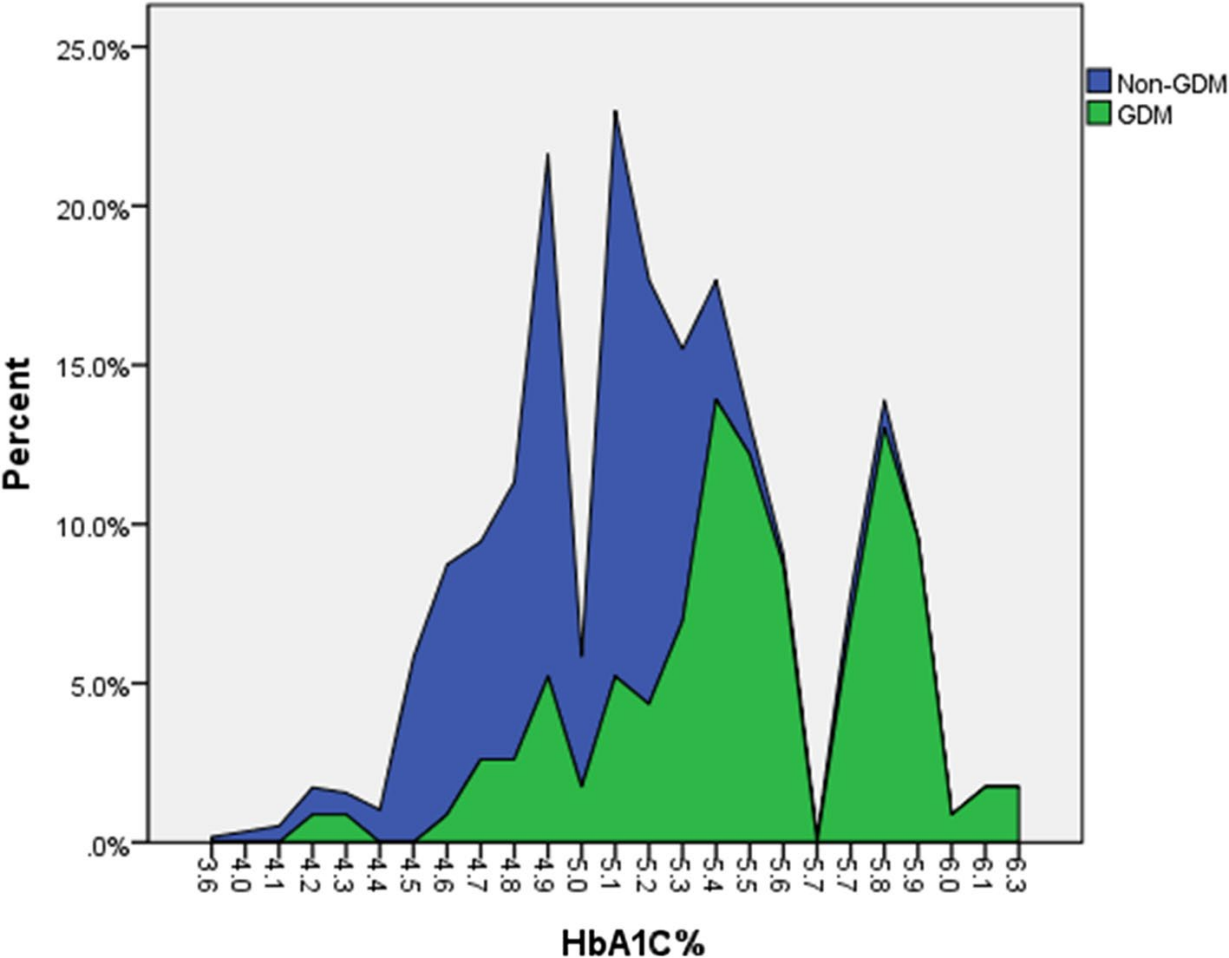
HbA1c distribution in women with and without gestational diabetes (GDM)

In pregnant women with GDM, the average HbA1c level was 5.45 ± 0.39% compared to 4.96 ± 0.30% in the women without GDM (*P* < 0.001). In addition, HbA1c overlap in women with and without GDM.s is depicted in Fig. 2. Use of HbA1c could decrease requesting OGTT in 40.4% of the pregnant women (28.70% with HbA1c < 4.85 and 11.7% with HbA1c ≥ 5.45%).

**Fig. 3:**
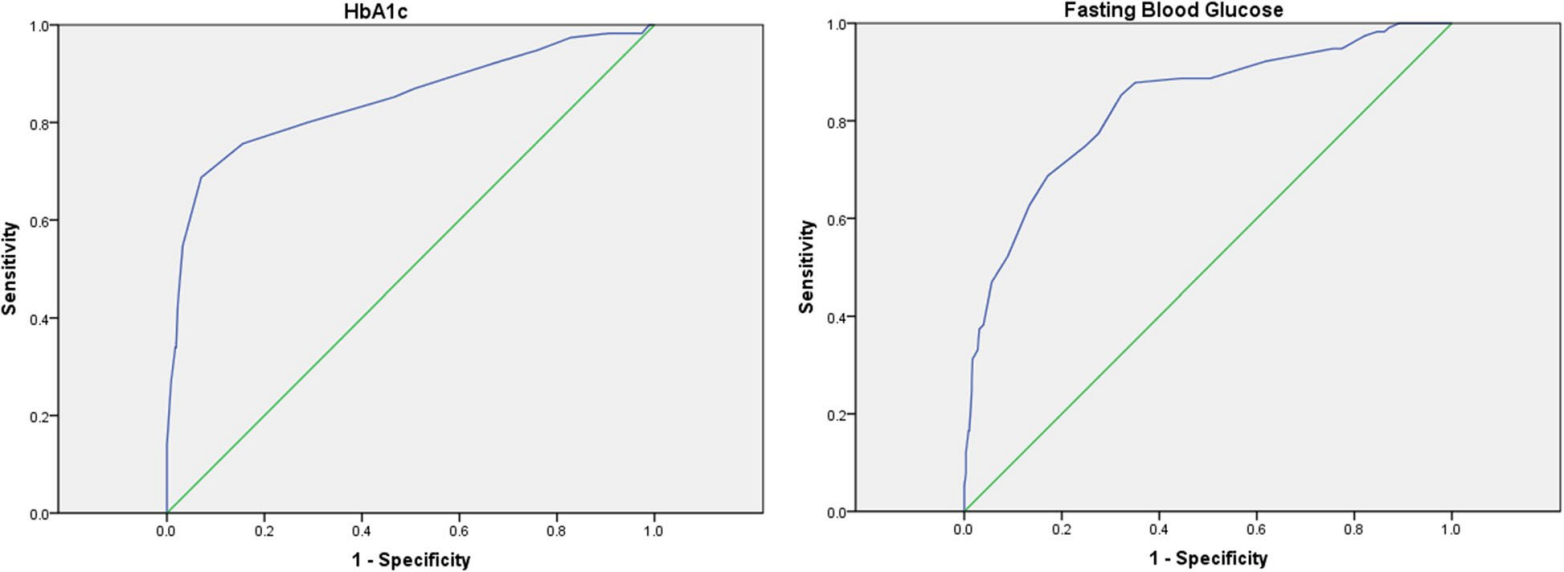
Receiver operating characteristic (ROC) curve for sensitivity and specificity according to different HbA1c and fasting blood glucose thresholds

The area under the ROC curve for diagnosing GDM by HbA1c was 0.84 (95% CI: 0.79–0.89; *P* < 0.001) (Fig. 3). The sensitivity and specificity for ruling out GDM at an HbA1c cut-off value of 4.85% was 92.2 and 32.8%, respectively, with a 95.5% NPV and a 21.2% PPV. Furthermore, sensitivity and specificity for diagnosing GDM at an HbA1c cut-off value of 5.45% was 54.8 and 96.8%, respectively, with a 91.5% NPV and a 76.8% PPV. The diagnostic profile of HbA1c is shown in Table 2.

**Table 2:** The diagnostic profile of HbA1c

HbA1c (%)	Sensivity	Specifity	NPV	PPV
2.60	100	0	–	16.4
3.80	100	0.2	100	16.4
4.05	100	0.5	100	16.4
4.15	100	1.0	100	16.5
4.25	99.1	1.9	91.6	16.5
4.35	98.3	2.6	88.2	16.5
4.45	98.3	3.6	91.3	16.6
4.55	98.3	9.4	96.4	17.5
4.65	97.4	17.3	97.1	18.7
4.75	94.8	24.1	95.9	19.7
4.85	92.2	32.8	95.5	21.2
4.95	87.0	49.2	95	25.1
5.05	85.2	53.3	94.8	26.4
5.15	80.0	71.1	94.7	35.2
5.25	75.7	84.4	94.6	48.8
5.35	68.7	93.0	93.7	65.8
5.45	54.8	96.8	91.5	76.8
5.55	42.6	97.8	89.6	79
5.63	33.9	98.1	88.3	78
5.68	33.9	98.3	88.3	79.5
5.75	27.0	99.1	87.3	86.1
5.85	13.9	100	85.5	100
5.95	4.3	100	84.1	100
6.05	3.5	100	84	100
6.20	1.7	100	83.8	100
7.30	0	100	83.5	–

*HbA1c* glycosylated hemoglobin, *NPV* negative predictive value, *PPV* positive predictive value

The area under the ROC curve for diagnosing GDM by FBG was 0.83 (95% CI: 0.78–0.87; *P* < 0.001) (Fig. 3). The diagnostic profile of FBG is shown in Table 3.

**Table 3:** The diagnostic profile of fasting blood glucose

Fasting blood glucose (mg/dl)	Sensivity	Specifity
58.00	100.00	0.00
62.00	100.00	0.17
65.50	100.00	0.34
66.50	100.00	0.68
67.50	100.00	0.85
68.50	100.00	1.03
69.50	100.00	2.22
70.50	100.00	5.64
71.50	100.00	6.32
72.50	100.00	9.23
73.50	100.00	10.77
74.50	99.13	12.82
75.50	98.26	13.85
76.50	98.26	15.38
77.50	97.39	17.95
78.50	94.78	22.56
79.50	94.78	24.44
80.50	93.04	33.68
81.50	92.17	38.29
82.50	88.70	49.57
83.50	88.70	55.56
84.50	87.83	64.96
85.50	85.22	67.86
86.50	77.39	72.48
87.50	74.78	75.21
88.50	68.70	82.91
89.50	62.61	86.67
90.50	52.17	91.11
91.50	46.96	94.36
92.50	38.26	96.07
93.50	37.39	96.92
94.50	33.04	97.26
95.50	31.30	98.29
96.50	26.96	98.46
97.50	25.22	98.46
98.50	21.74	98.63
99.50	16.52	98.97
100.50	16.52	99.15
101.50	12.17	99.66
102.50	7.83	99.66
104.00	5.22	100.00
106.00	2.61	100.00
112.00	1.74	100.00
118.00	0.00	100.00

## Discussion

The prevalence of GDM in our study was 16.4%, which was in reported ranges of HAPO Study Cooperative Research Group [3], but it was higher compared to some former studies [5, 16]. This variation is because of referring high-risk pregnant women to our hospital, using different GDM diagnostic thresholds, and different screening OGTTs in studies. For instance, we used the Carpenter-Coustan threshold, which has a lower threshold for the GDM detection compared to the National diabetes data group that applied in Benaiges et al. study [5].

In this study, we found that the average first-trimester FBG and HbA1c of GDM women were significantly higher compared with normoglycaemic women, which was similar to previous studies [5, 17].

The women with HbA1c greater than 6% were at a higher risk for GDM, and those with HbA1c less than 4.5% were not complicated by GDM. Although in our study, HbA1c in GDM women fell within the range of former studies, HbA1c in normoglycaemic women was lower the average HbA1c concentration reported in Asian Indian pregnant women, 5.36 ± 0.36% [18, 19]..

This study has shown excellent reliability of HbA1c for the GDM diagnosis with an AUC of 0.84. This finding was in line with some previous studies [20–22] such as a recent meta-analysis evaluating 6406 pregnant women [23] in which the AUC values ranged from 0.80 to 0.93 and in contrast with the other ones [12, 24–28].

GDM has public health implications and the early detection of it is clinically essential. Women with GDM are at risk for developing type 2 diabetes mellitus and impaired glucose tolerance and have a higher risk for cardiovascular diseases along with their life [16].

The appropriate test for GDM diagnosis is a test with a high sensitivity to diagnose the patients and high specificity, but this could not be observed in the HbA1c test. As found in our study, by increasing HbA1c, the sensitivity decreased, and the specificity increased. Although using the higher 5.45% and lower 4.96% cut-off values for HbA1c could help, distinguish the pregnant women with a higher and lower risk for GDM, respectively [29].

Despite the acceptance of HbA1c among pregnant women and its advantages over other GDM diagnostic methods, such as its less intra-individual coefficient of variation of 1.9 to 4.2% [30], as our study demonstrated, HbA1c had a significant overlap in both normal and GDM groups.

Another challenge with HbA1c is its significantly decreasing in pregnancy, a decline of the upper normal level of HbA1c from 6.3 to 5.7% in early pregnancy and to 5.6% in the third trimester of pregnancy, indicating a reduction of HbA1c during normal pregnancy that is of clinical importance when defining the goal for HbA1c during pregnancy complicated with diabetes [31].

Furthermore, physiological hydremia during pregnancy, anemia, slower intestinal transition, increased red cell turnover, and nutritional alternations are factors that can considerably affect the HbA1c value [32]. For these reasons, there is no guideline using HbA1c for the diagnosis of GDM.

The advantage of our research was the large sample. Furthermore, all information was gathered from the same lab and the same clinic. However, our study had some limitations. For instance, we did not evaluate the cost-saving potentials while using HbA1c for screening GDM. In addition, there are possible variations in the degree of HbA1c, independent of glycemia, which could be associated with family history or genetics. In this study, we did not include these factors.

## Conclusion

It seems that the first-trimester HbA1c, because of its insufficient sensitivity or specificity, cannot replace OGTT for the diagnosis of GDM. However, women with higher first-trimester HbA1c have a high risk for GDM.

## Data Availability

The datasets used and/or analyzed in this study will be made attainable from the corresponding author upon request.
